# Robotic assistance for skull base biopsy: a feasibility study in phantom and cadaver

**DOI:** 10.3389/fonc.2025.1669974

**Published:** 2025-09-10

**Authors:** Jian-Hua Zhu, Xiao-Jing Liu

**Affiliations:** ^1^ Department of Oral and Maxillofacial Surgery, Peking University School and Hospital of Stomatology, Beijing, China; ^2^ National Center for Stomatology & National Clinical Research Center for Oral Diseases & National Engineering Research Center of Oral Biomaterials and Digital Medical Devices& Beijing Key Laboratory of Digital Stomatology & NHC Key Laboratory of Digital Stomatology & National Medical Products Administration (NMPA) Key Laboratory for Dental Materials, Beijing, China

**Keywords:** robot, skull base, navigation, biopsy, precision

## Abstract

**Objective:**

Biopsy of suspicious lesions in deeply situated target areas is the first step in clinical management. This study aims to investigate the feasibility and accuracy of a novel robot system for skull base biopsy guided by Cone beam CT (CBCT).

**Materials and methods:**

The skull phantom and cadaveric specimen were used for tests. The biopsy were performed by a custom 7 degrees of freedom robot system. CBCT images were used for planning trajectories and the data were sent to the robot control unit. Following registration, the puncture needle was automatically inserted into the target by the robot guided by navigation. Location deviation was instantly calculated and the result was verified after postoperative image scanning.

**Results:**

All 20 interventions were successfully performed in phantom and cadaver respectively. In phantom experiments the mean placement error was 0.56± 0.21 mm (measured by the navigation system) vs. 1.77 ± 0.13 mm (measured by image fusion) (*P*< 0.001); in cadaveric studies the corresponding figures were 0.71 ± 0.15 mm vs. 3.10 ± 0.18 mm (*P<* 0.001). Accuracy was better in the phantom experiment (*P<* 0.001). The Pearson Correlation Coefficients (*r*) was 0.639 and 0.723 in phantom experiments and cadaveric studies respectively.

**Conclusion:**

The performance of robot-assisted skull base biopsy is feasible and accurate. Clinical tests will need to be demonstrated in further studies.

## 1 Introduction

Biopsy of suspicious lesions remains a standard requirement for better counseling and therapy in clinical management (1). Deep lateral facial/skull base lesions are diverse, rare, and surgically challenging. Conventionally, the open biopsy is performed to obtain a sufficient amount of specimen for diagnosis. The deep location and complex anatomy of skull base lesions (e.g., mandibular barrier, neurovascular risks) render open biopsy as high-risk as resection. This, combined with contraindications in patients unsuitable for invasive procedures (due to comorbidities, preference for non-surgical treatment, or refusal of surgical morbidity), limits its application.

With the development of digital surgical techniques, the navigation-guided core-needle biopsy is recommended as an excellent technique in the diagnosis of skull base masses, but it much still depends on the surgeon’s experience and hand-eye-mind coordination (1, 2). For percutaneous interventions, the manually locating the skin entry sit, adjusting the angulation of the needle and eluding the osseous intervention can all be technically challenging for the surgeon (3). Poor accuracy may results in damage to surrounding normal tissue and increase the risk of recurrence because of tumor seeding. Besides, the navigation surgery needs repeatedly switch vision between the patient and the monitor, while the integration of imaging and robotic technology can act as a “third hand and eye” for the surgeon regardless of tremor, fatigue and the risk of exposure to radiation as we reported (3–5). This technique has the potential to improve the success rate of tumor biopsy sampling in clinical practice. However, the previous 5 degrees of freedom robot device is an arch-like structure, which was fixed rigidly on operating table with a limited three-dimensional workspace. Furthermore, to keep the spatial relationship stable during the operation, the skull model was rigidly fixed with a head clamp during operation in case of the accidental motion of the head, which seemed to be clumsy in reality. On the base of the first stage of research and question, We have developed a novel 7 degrees of freedom robot system for percutaneous needle biopsy in skull base region, and in this article we evaluated the feasibility and reliability of this robot system in phantom and cadaveric studies, which is the necessary component for introducing it into clinical practice.

## 2 Material and methods

### 2.1 Experimental setup

**Figure 1:**
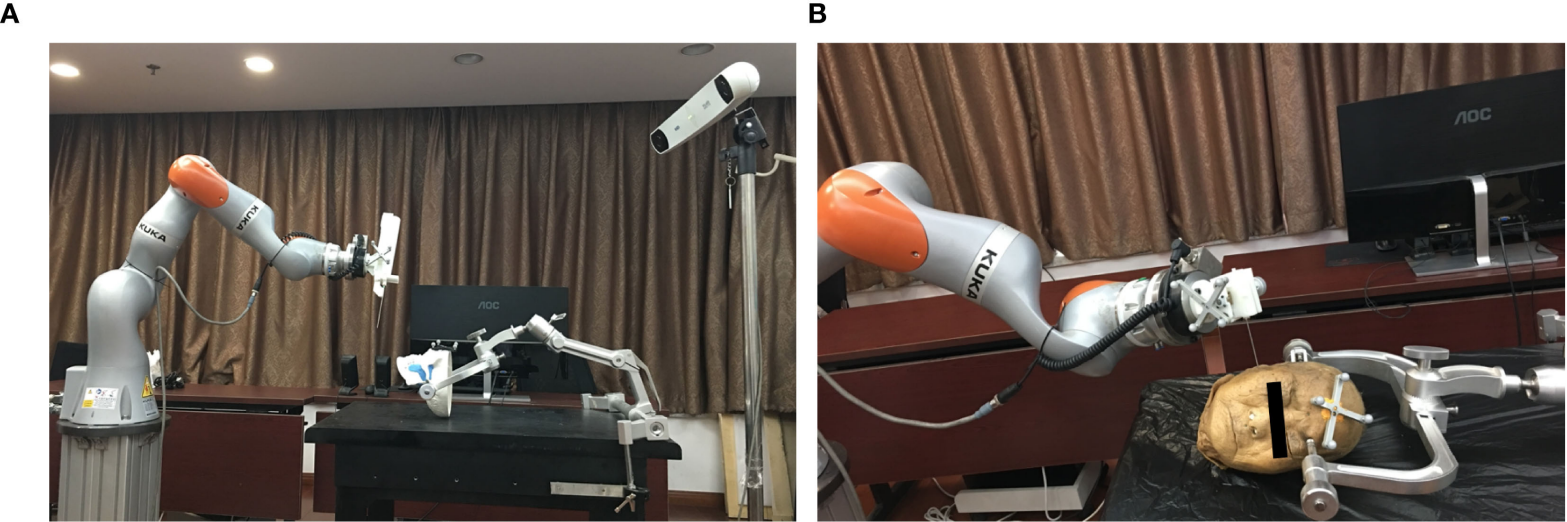
Overview of the robot system with 7 DOF: the phantom study **(A)**, the cadaver study **(B)**.

The robot device comprises a light structure with an end effector for needle orientation (**Figure 1**). The technical specifications of the novel robot system include the following: 1) a 7-degrees of freedom robot arm (iiwa 14, KUKA, Germany), which is sensitive and has a ±0.15mm repositioning accuracy with fast response speed to guarantee the accuracy and improve the safety. Besides, it’s all-aluminium outer casing greatly reduces the weight, which makes it convenient to move in the operating room; 2) an optical tracking system (Polaris; Northern Digital Inc., Waterloo, Canada) with 0.25-mm positioning accuracy and 20-Hz update rate; 3) a 6-dimension force sensor (Delta IP60; ATI Industrial Automation, USA) placed between the joint and the end effector; 4) end effector for clamping biopsy gun and needle; 5) an open source robot operating system for robot control, workflow tracking and safety guarantee; 6) a custom GUI for preoperative surgical planning and registration, intraoperative real-time navigation and postoperative validation.

As we previous reported (3, 6), in the phantom study, a synthetic human skull (A150; Kexin Scientific Equipment, Zhangjiagang, China) was used with plasticine^®^ placed around the skull base to imitate soft tissue. Oval masses of 3cm diameter were placed beneath the skull base to act as the target “tumours”. In the cadaveric study, a formalin-preserved human head were provide by the anatomy department of our institute. Iopamidol (370 mg/mL; Bracco Sine Limited, Shanghai, China) was injected into skull base making the tissues containing the injected dye acted as the “tumours”. The study was carried out in accordance with The Code of Ethics of the World Medical Association (Declaration of Helsinki) and approved by the local ethics committee(PKUSSIRB201626010).

### 2.2 Workflow

#### 2.2.1 Data acquisition

Preoperative CBCT image data was obtained from a NewTom VG scanner (Quantitative Radiology, Verona, Italy). The CBCT data (field-of-view 15cm × 15cm, matrix 512 × 512, slice thickness 0.3mm) was transferred to the host computer in DICOM (Digital Imaging and Communications in Medicine) format and displayed on a custom graphical user interface (GUI) for the surgical planning and registration.

#### 2.2.2 Trajectory planning

**Figure 2:**
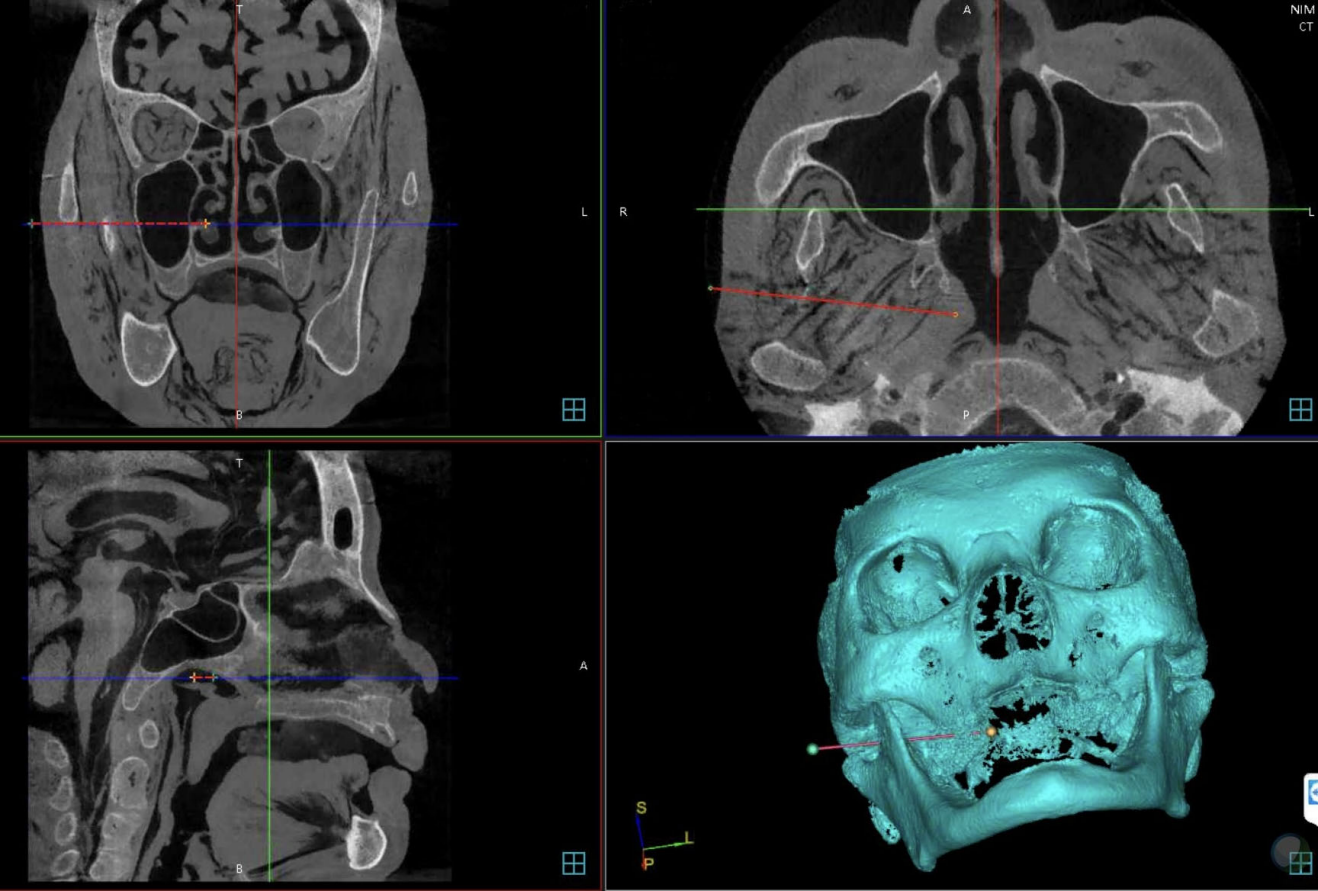
Needle trajectory planning.

The boundary of the “tumour” was manually segmented and the three-dimensional reconstruction was applied for the skull. The target point and the skin entry point was subsequently selected on the GUI to define the optimal needle trajectory automatically with allowance made for manual adjustment (**Figure 2**). After the planning process, the relevant data were calculated and sent to the robot controller by socket communication through the local area network.

#### 2.2.3 System registration

The registration of whole robotic surgery system includes four coordinate spaces: robot space, patient space, optical tracking space and image space, which should be aligned by matrix transformation. LAN-based socket networking linked the GUI with the robot controller on a remote console. As an intermediate coordinate, the optical tracking system was used to correlated the different coordinate systems. Firstly, nine titanium screws of 2-mm diameter (Synthes, Solothurn, Switzerland) were inserted around the craniomaxillofacial region to act as fiducial markers follow the advice given by Caversaccio (6). A none-invasive dynamic registration reference was used to rigidly fix reference base on forehead area to allow the navigation system track the position of the head in real-time. The coordinates of fiducial markers on the skull could be obtained by the optical probe of the navigation system while the corresponding data on the image were manually selected on the GUI. Subsequently, the registration of the skull and the images to the navigation system was accomplished through the fiducial markers by means of an improved iterative closest point (ICP) algorithm (7). Secondly, the registration of the robot to the tracking system was also performed by improved linear rotation calibration method (8); the location error of the robot’s movements guided by the navigation system was permissible 0.25mm (8). As a result, the images, patient, optical navigation and robot were then aligned by matrix transformation. Th motion error was verified by a calibrated standard model with visual feedback after each registration.

#### 2.2.4 Needle positioning

Once the surgical planning was confirmed by surgeon, the relevant intraoperative needle orientation data were calculated and sent to the robot controller. Although the 16-gauge needle (Bard Peripheral Vascular, Inc., Arizona, USA) could be driven to the target position automatically by the robot arm along the planned trajectory, the interactive control system was designed as a “surgeon-in-closed-loop” mode for safety consideration. On the one hand, the needle would be further advanced only after receiving confirmation from the surgeon when the needle was at the entry point, otherwise it would remain motionless. Besides, an emergency switch would be pressed by surgeon to keep the robot system stationary within 0.5 seconds a if an accident happened. On the other hand, the real-time visual feedback was displayed on the GUI through continuous updating of the needle position data acquired by the optical tracking system. Finally, as an alerting, continuous axial force acquired by force sensor was synchronously displayed on the GUI after signal translation in case of the accidental collision of the needle with bone during intervention process. 30 N axial puncture force was the security threshold during the surgery for an emergency response as we previous recommended to avoid accidental collisions with skull (5).

#### 2.2.5 Postoperative verification

**Figure 3:**
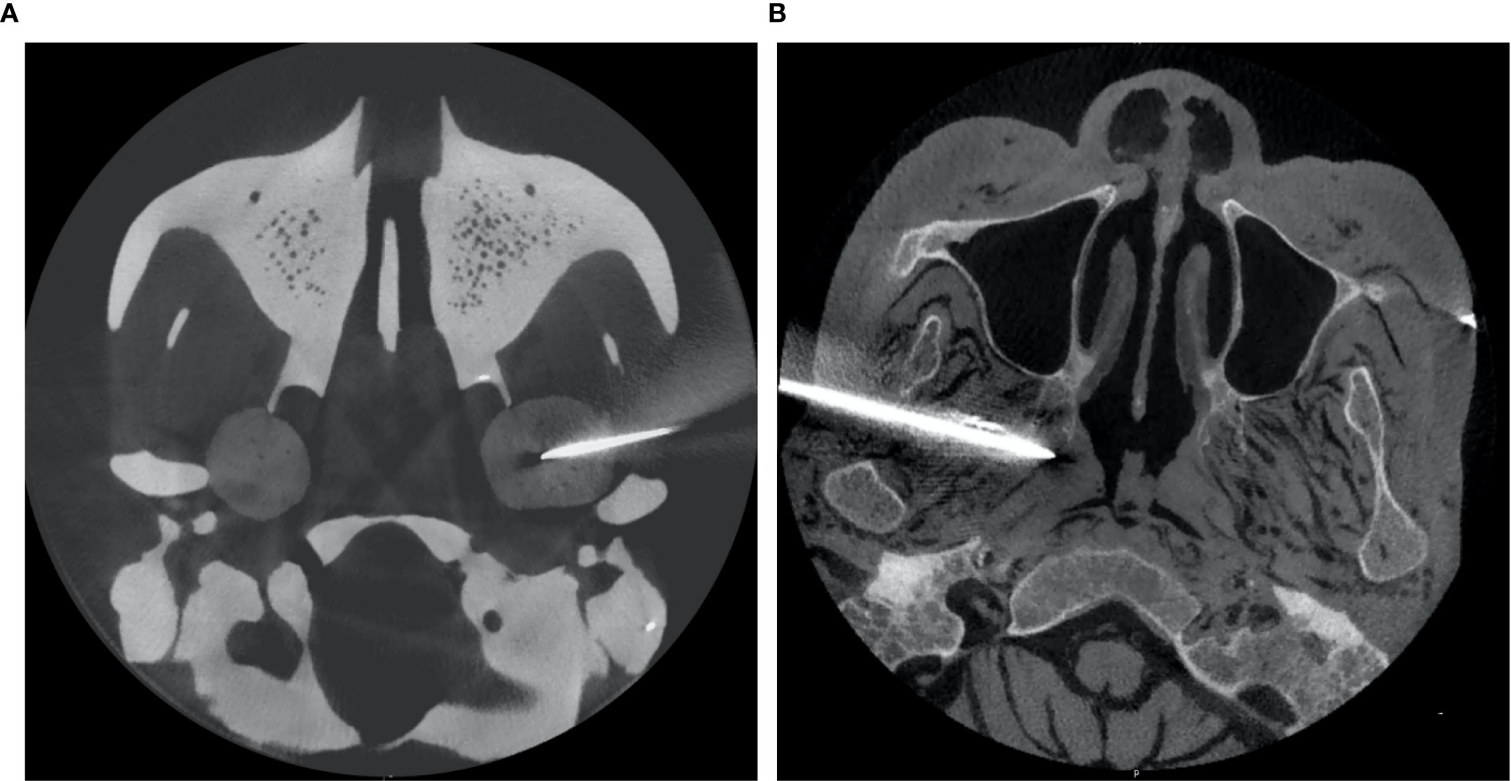
Postoperative images of the experiment: the phantom study **(A)**, the cadaver study **(B)**.

**Figure 4:**
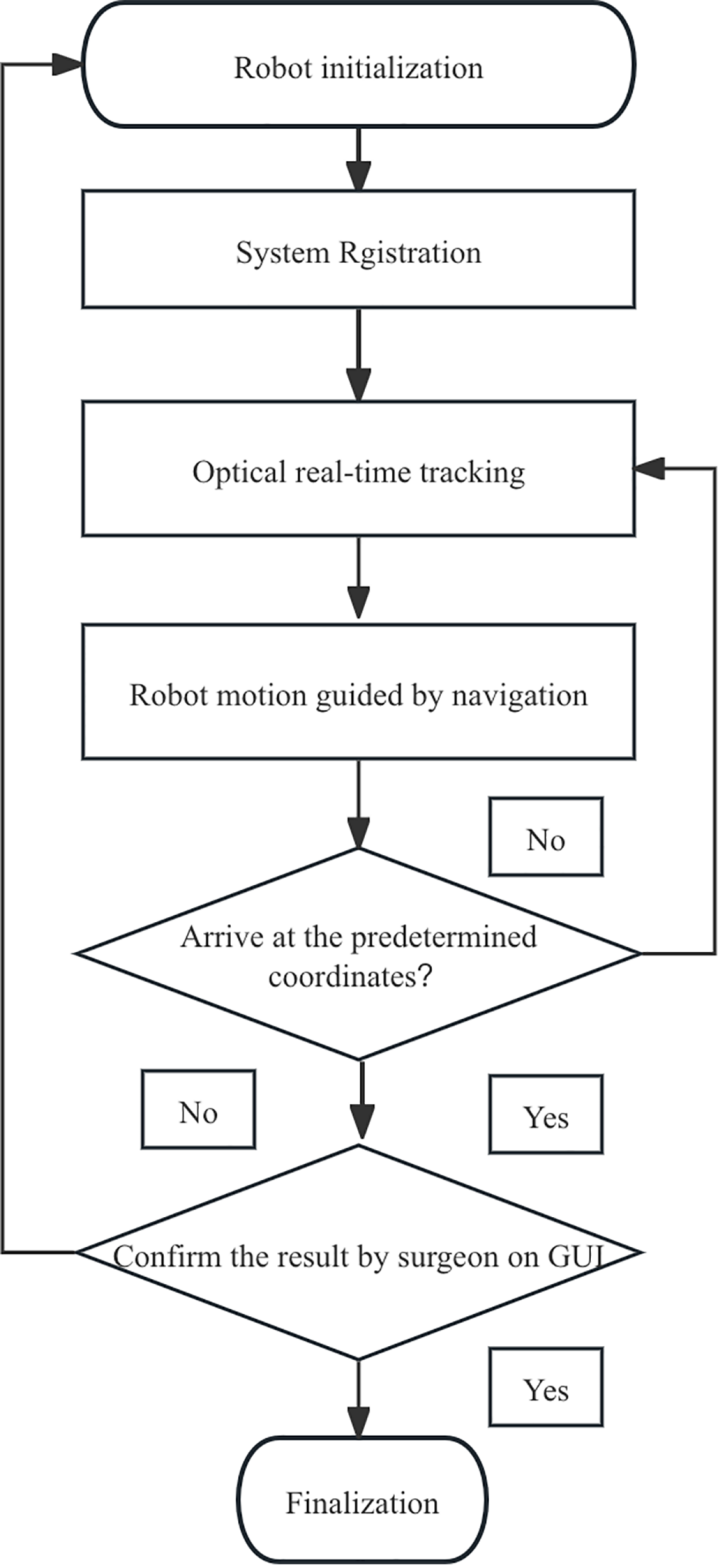
The workflow diagram of robot-assisted puncture surgery.

Once the intervention procedure was completed, the instantaneous data of needle orientation acquired by the navigation system was sent back to the GUI for accuracy verification. Subsequently, postoperative CBCT scanning was performed to re-verify the position of the needle tip and its trajectory (**Figure 3**). The preoperative planning coordinates (X1, Y1, Z1) and the postoperative (X2, Y2, Z2) needle position were aligned by matrix transformation after image fusion; as the gold standard,the total error was defined as the Euclidean distance $\left(\sqrt{\Delta {\text{X}}^{2}+\Delta {\text{Y}}^{2}+\Delta {\text{Z}}^{2}}\right)$ calculated by the offsets of the coordinates. The workflow was shown in **Figure 4**.

#### 2.2.6 Statistical analysis

Statistical analysis was performed using IBM SPSS, Version 24 (IBM Corp., Armonk, NY, USA). The Shapiro-Wilk and Bland-Altman plots were use to demonstrate homogeneity. The Whitney U test was used to compare the accuracy of puncture accuracy measured by the navigation system and by postoperative verification. The Wilcoxon Rank Sum test was used to compare the puncture accuracy and depth between phantom experiments and cadaveric studies. Statistical significance was set at *P* ≤ 0.05. The Pearson Correlation Coefficients (*r*) was used to analyze correlation between the puncture accuracy and insertion depth in phantom experiments and cadaveric studies.

## 3 Results

**Figure 5:**
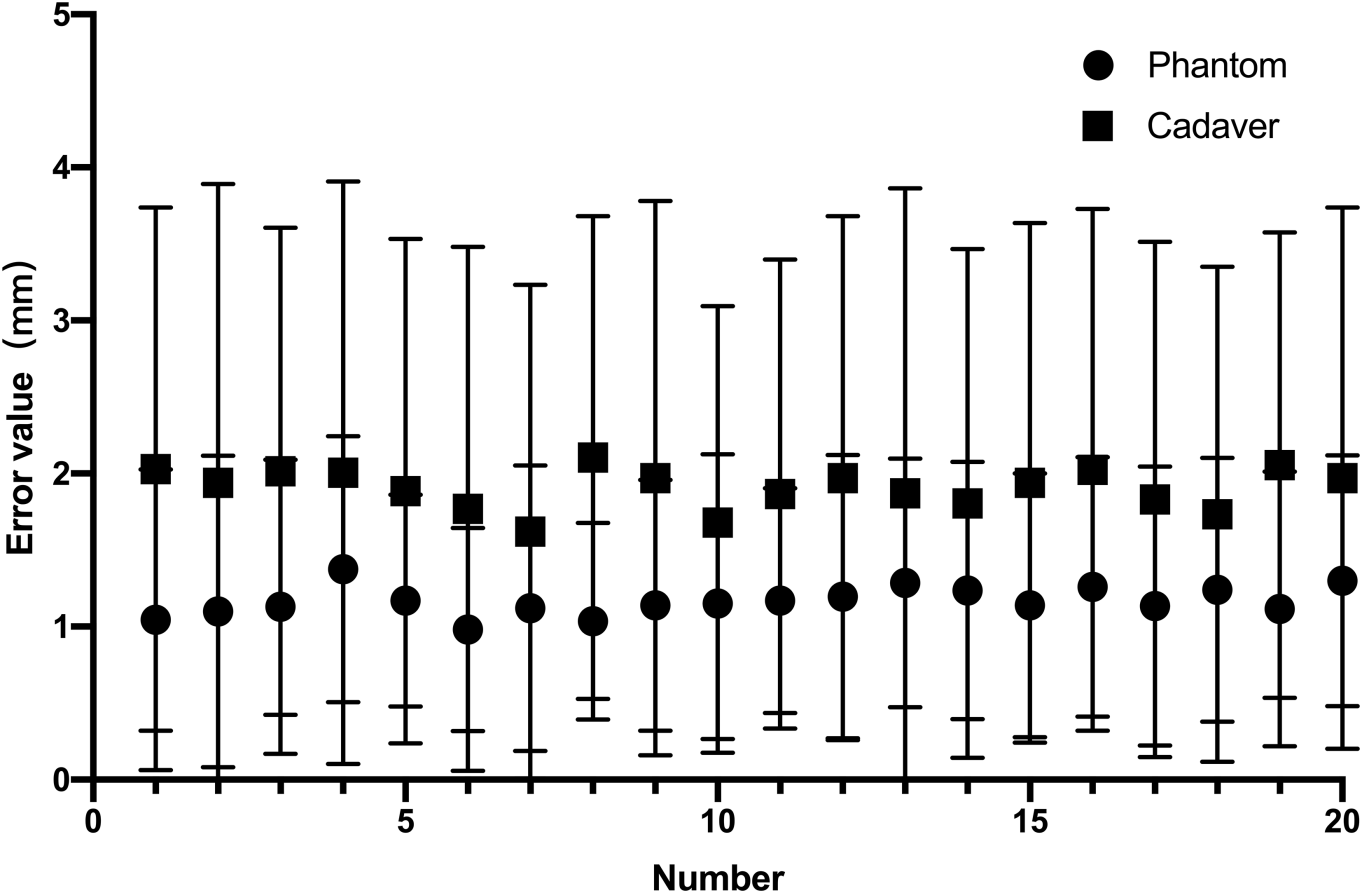
The needle insertion error measured by the navigation system and by image fusion in phantom experiment (0.56± 0.21mm vs. vs. 1.77 ± 0.13mm) and the cadaveric study(0.71 ± 0.15mm vs. 3.10 ± 0.18mm).

**Figure 6:**
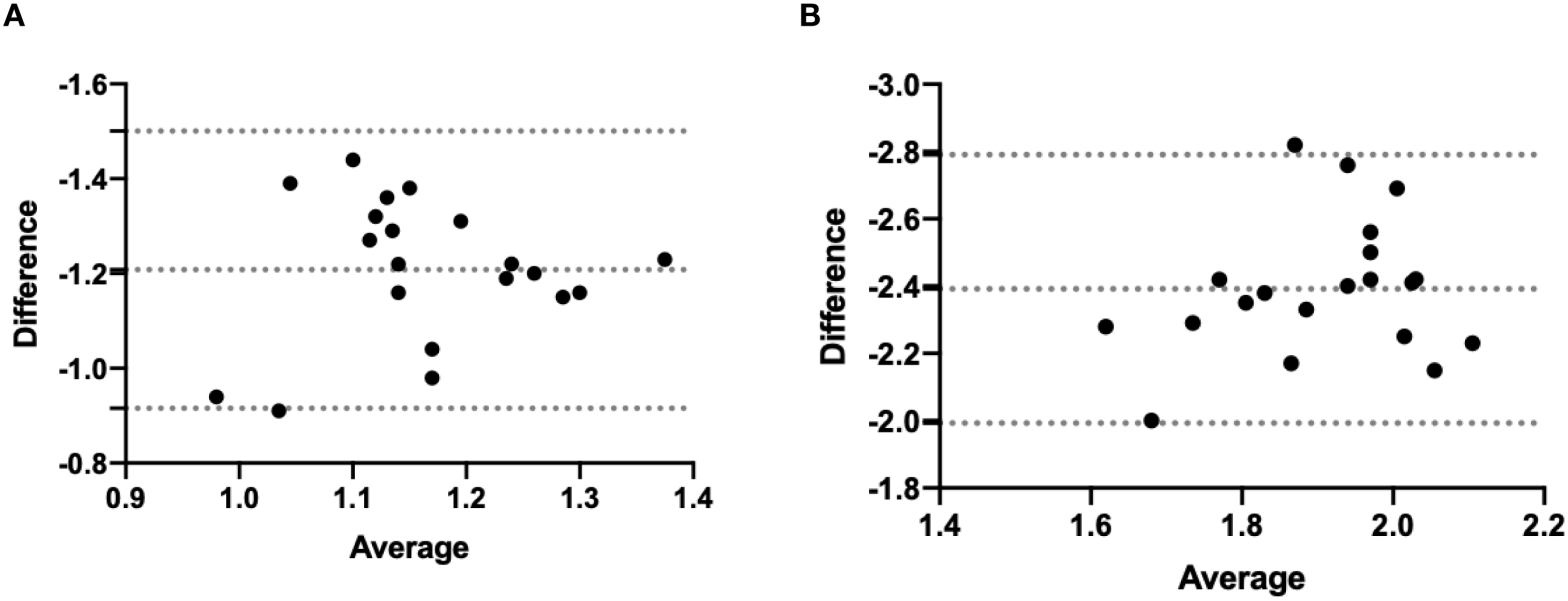
Bland-Altman of two accuracy measurement in phantom experiment **(A)** and the cadaveric study **(B)**.

All 20 interventions were successfully performed in a phantom sample and a cadaver sample respectively (**Figure 5**). The mean deviation of the needle tip measured by the navigation system and by image fusion were shown in **Table 1**. The Shapiro-Wilk was *P* = 0.767, 0.025, 0.840, 0.173 respectively with accuracy measured by navigation system and by postoperative verification in phantom and cadaver. The Bland-Altman plots with 95% Confidence Interval for Mean were shown in **Figure 6**. There were statistically significant difference both in phantom (*Z*=-3.921, *P*<0.001) and in cadaver (*Z*=-3.921, *P*<0.001). The interventions was more accurate in the phantom experiments than in the cadaveric studies (Z=-5.412, *P*<0.001) at a comparable insertion depth (Z=-1.611, *P* = 0.107). Pearson correlation coefficients (*r*) was 0.639 and 0.723 in phantom experiments and cadaveric studies respectively.

**Table 1:** Accuracy and insertion depth of robot-assisted needle intervention.

Method	Accuracy (mm)		Depth (cm)
	Measured by navigation system	Measured by image fusion	Mean± Standard deviation (Minimum, Maximum)
	Mean± Standard deviation (Minimum, Maximum)	Mean± Standard deviation (Minimum, Maximum)	
Phantom	0.56± 0.12 (range: 0.35-0.76)	1.77 ± 0.13 (range: 1.45-1.99)	5.09 ± 0.12 (range: 4.85-5.26)
Cadaver	0.71 ± 0.15 (range: 0.49-0.99)	3.10 ± 0.18 (range: 2.68-3.35)	5.16 ± 0.13 (range: 4.89-5.36)

## 4 Discussion

Traditionally, maxillofacial surgeries required large incisions due to the complex anatomy and restricted operating space which causing the patient to suffer from serious psychological problems. Advances in minimally invasive techniques improved functional preservation, reduced complications, and enhanced outcomes. This drives rising adoption of robotic maxillofacial surgery, including transoral procedures and dental implants (9, 10). Robotic surgery entered head and neck practice in 2005 (vallecular cyst removal), building on its 1988 neurosurgical debut in brain biopsies (10). The approach still faces significant challenges in anatomically intricate zones such as the skull base. Hence in deeply situated target areas, pathologic diagnosis is the first step for these lesions in clinical management.

Nevertheless, the robotic system’s usability and interference resistance significantly influence preoperative setup time, intraoperative efficiency, and clinician training expenditures, all of which contribute to total surgical costs. Hence a serial-architecture collaborative robot was chosen to ensure scalability and operational flexibility. Intraoperatively, real-time optical tracking of the robot’s end-effector and patient ensures the safety, accuracy, and efficiency of percutaneous interventions. Although no standard for position accuracy has been described for routine clinical practice and surgeons define the error threshold according to the individual case (5). Safety remains the top priority in robot-assisted surgery to avoid serious complications like unintended hematomas or nerve damage. Compared with the reported frame-based robot (5), the novel 7-degrees of freedom robot with mobile light structure achieving a repeatability of ±0.15mm showed similar precision (1.73± 0.60mm versus 1.77 ± 0.13mm in phantom)but providing enhanced stability, flexibility and workspace. Although the low data acquisition frequency (20 Hz update rate) and the problem posed by the line of sight between the camera and the infrared markers are non-negligible drawback with optical navigation (11), it has been actually used in clinical interventions as it provides real-time vision feedback by continuous updating of the instrument position. Higher frequency sensors should be developed in the future. Since surgical space calibration accuracy directly impacts overall system precision, this study introduced an improved automatic calibration algorithm for linear rotation, and the experimentally verified average errors of robot end-to-end position and posture guided by navigation were 0.25mm and 0.2mm, respectively (8). The system puncture accuracy of the needle tip location guided by the navigation consisted of registration errors (0.1 to 1.8mm) (6), optical localizer errors (0.35mm), motion control algorithm and needle deflection. Although the close-loop control strategy with submillimeter was performed like previous studies during needle intervention, inherent systematic errors cannot be entirely eliminated. In reality, it is hardly practical to separate the contribution of each component, and to quantify them for every time especially for needle deflection. Besides, the proposed algorithm effectively compensates for manual calibration inaccuracies and system noise. Actually the total error is not just a sum of the errors of each system components such as registration errors, optical localizer errors, image distortion and human error, it even exceeds the summation because of needle deflection. The difference of the accuracy measured by the two methods was statistically significant both in the phantom and cadaveric study (*P<* 0.001). The navigation error of the entire robotic system should bear primary responsibility for this outcome, given the material homogeneity of the Plasticine^®^. In line with predictions, phantom trials showed significantly greater needle intervention accuracy than cadaveric studies (*P*<0.001) for matched insertion depths. A plausible explanation is that needle deflection constituted the dominant factor contributing to needle displacement in biological tissues, consistent with prior reported findings (3, 12–14). The Pearson correlation coefficient is a statistical measure used to quantify the linear correlation between two variables. Its value ranges from -1 to +1, indicating both the direction (positive/negative) and strength of the relationship. Notably, Pearson correlation analysis demonstrated that insertion depth more significantly influenced puncture accuracy in cadaveric tissues (*r* = 0.723) than in phantom models (*r* = 0.639), possibly due to the heterogeneous mechanical properties of biological specimens. The accuracy of 3-6mm *in vivo* is estimated with manual needle placement as reported (15), and The mean accuracy was measured in cadaver 3.10mm is clinically acceptable compared with manual procedure. The precision data obtained from phantom and cadaver studies suggest that maintaining a 5mm safety margin during needle path planning helps prevent injury to vital neurovascular structures and ensures containment within tumor margins.

To reduce targeting errors and enhance precision in needle insertion procedures, researchers have conducted comprehensive studies on both animal and human organ punctures, as well as percutaneous interventions, through multiple approaches (16–18): 1) Viscoelastic Tissue Modeling – Developing biomechanical models to characterize tissue deformation under needle insertion forces; 2) Tissue Force Deformation Mechanisms – Investigating how biological tissues respond to mechanical stresses during penetration; 3) Force Analysis During Insertion – Measuring and analyzing real-time interaction forces between needles and tissues; 4) Needle Deflection Behavior – Studying bending patterns and trajectory deviations of flexible needles in heterogeneous tissues. Mahvash et al. (19) conducted experiments using fresh bovine hearts, demonstrating that needle insertion-induced tissue rupture generates propagating cracks, which cause tissue deformation and subsequent needle trajectory deviation, ultimately reducing targeting accuracy. Their findings indicate that higher insertion speeds minimize crack formation and propagation, thereby improving precision (19), but low data acquisition frequency of navigation imposes an upper bound on needle insertion speeds. Engh et al. (20) demonstrated that needle rotation can reduce targeting error, which showed that incorporating rotational degrees of freedom during insertion with a bevel-tipped needle significantly decreases deflection, establishing that increased needle rotation correlates with improved insertion accuracy. However, rotational insertion in the tightly spaced, neurovascularly dense skull base heightens risks of traumatic complications including hematomas and neural trauma. Li et al. investigated the effect of needle tip geometry on the needle deflection and results showed that multi-bevel needle tip geometry with the tissue separation point below the needle groove face may reduce the needle deflection (21). Critically, tissue heterogeneity and inter-patient variability challenge the predictive accuracy of biomechanical models for preemptive error compensation in needle insertions. Robotic steering of bevel-tip needles primarily employs intermittent axial rotation to realign the bevel orientation, thereby correcting needle trajectory and is feasible for clinical use. To address the problem and automate needle insertion, robotic systems, Lehmann et al. developed mathematical models for estimation and prediction and control algorithms by introducing lateral force-based needle steering method complementing axial rotation and this approach enabled continuous deflection control (22).

Unfortunately, due to limitations in robotic degrees of freedom, in previous studies the head should keep a specific fixed position with a head clamp during operation. This poses several intraoperative challenges including: 1) dynamic surgical space alterations due to evolving procedural demands, 2) optical tracker pose displacement from collisions mandating recalibration because of limited workspace, and 3) calibration failures from robotic occlusion, necessitating manual pose re-alignment. These factors elevate procedural complexity and planning workload. The present studies represent significant advancements compared to earlier methods in improving efficiency, which was a dynamic registration frame rigidly fixed on the patient head to track the position of the head in real-time as used in the currently routine clinic. The head motion was limited in the study because of the limitation of the phantom and cadaver. In clinic, rapid movements of the head should be avoided as much as possible because of low data acquisition frequency sensors. Recalibration was not required provided that the pose of the digital reference frame is unchanged since it could make immediate compensate for the programmable trajectory. While current robotic systems have made significant strides in clinical translation compared to prior research, this study has several important limitations worth addressing. Firstly, as a preliminary investigation using phantoms and formalin-fixed cadavers, it did not account for complex anatomical structures like carotid arteries or neural tissues. As demonstrated by Ling et al. (23) formalin fixation increases tissue elasticity by 120.2% compared to fresh samples, underscoring the necessity of fresh-tissue validation in our study. While cadaveric models provide foundational validation, future work requires testing in perfused fresh tissues and survival animal studies to quantify dynamic tissue effects. Secondly, Given the poor clinical acceptability of invasive registration methods, significant efforts must be directed toward developing non-invasive alternatives that maintain targeting accuracy while improving patient comfort and procedural efficiency. Although noninvasive navigation methods (e.g., point-based registration with dental arch-mounted fiducials or surface matching) show clinical promise, their registration accuracy with robotic systems requires rigorous verification before replacing current invasive registration approaches. The lastly, The robotic system necessitated a 30-minute preoperative phase for accurate multi-modal registration (patient-imaging-navigation-robot alignment), while procedural duration decreased significantly with surgical team experience. Robot-assisted puncture does not significantly increase time, cost and radiation exposure compared to current navigation-only biopsy, as the robot registration can be completed within 10 minutes. The workflow integration and training requirements is similar to the existing navigation surgery as we routinely performed. According to “The GAMER Statement—Reporting guideline for the use of Generative AI tools in Medical Research” (24), The author(s) declare that no Generative AI was used in the creation of this manuscript as shown in **Table 2**.

**Table 2:** The GAMER checklist.

No.	Item	Reported page
1	Did you use any GAI tools (such as large language models or large visual models) in any section or step of this manuscript or study?	□Yes ☑No □N/A
2	Specify the GAI tool(s) used, their versions and/or release dates and the date(s)/period the tools were used.	□Yes □No ☑ N/A
3	Describe whether a specific prompting technique was used to generate any content of the manuscript or to perform analyses during the study. Please also provide the unedited responses to the prompts.	□Yes ☑ No □ N/A
4	If a new GAI tool was developed or fine-tuned based on an existing AI model, report the name and version of the original model.	□Yes □No ☑ N/A
5	Describe the role of GAI tools in all phases of this study where they were used (including manuscript writing).	□Yes □No ☑ N/A
6	Report the specific section or paragraphs of the manuscript that GAI tools contributed to.	□Yes □No ☑ N/A
7	Describe how the content generated by GAI tools was verified and (when necessary) modified.	□Yes □No ☑ N/A
8	Describe how data privacy and confidentiality were ensured during the use of GAI tools.	□Yes □No ☑ N/A
9	Describe whether and how the use of GAI tools may have influenced the interpretation of results, the study’s overall accuracy, or conclusions.	□Yes □No ☑ N/A

AI, artificial intelligence; GAI, generative artificial intelligence; GAMER, Generative Artificial intelligence tools in MEdical Research; N/A, not applicable.

## 5 Conclusion

We developed a novel robotic system for percutaneous interventions and validated the accuracy and feasibility of its CBCT based optical navigation system. The bending of the puncture needle has a significant impact on the accuracy of the surgery. Preclinical results demonstrate that the system is efficient, reliable, and safe, while offering enhanced flexibility and a more unconstrained workspace compared to prior approaches.

## Data Availability

The original contributions presented in the study are included in the article/supplementary material. Further inquiries can be directed to the corresponding author.
